# Urinary SARS-CoV-2 RNA is An Indicator For The Progression and Prognosis of COVID-19 Disease

**DOI:** 10.21203/rs.3.rs-203728/v1

**Published:** 2021-02-18

**Authors:** Maoqing Tian, Lu Zhang, Kai Zhu, Bo Shen, Gang Wang, Yuan Song, Cheng Chen, Wei Liang, Yang Guan, Guohua Ding, Tiechi Lei, Xiaogang Li, Jingyuan Xie, Yongqing Tong, Huiming Wang

**Affiliations:** Renmin Hospital of Wuhan University: Wuhan University Renmin Hospital; Renmin Hospital of Wuhan University: Wuhan University Renmin Hospital; Renmin Hospital of Wuhan University: Wuhan University Renmin Hospital; Renmin Hospital of Wuhan University: Wuhan University Renmin Hospital; Guangzhou Military Command General Hospital: People's Liberation Army General Hospital of Southern Theatre Command; Renmin Hospital of Wuhan University: Wuhan University Renmin Hospital; Renmin Hospital of Wuhan University: Wuhan University Renmin Hospital; Renmin Hospital of Wuhan University: Wuhan University Renmin Hospital; Renmin Hospital of Wuhan University: Wuhan University Renmin Hospital; Renmin Hospital of Wuhan University: Wuhan University Renmin Hospital; Renmin Hospital of Wuhan University: Wuhan University Renmin Hospital; Mayo Clinic Rochester: Mayo Clinic Minnesota; Shanghai Jiaotong University: Shanghai Jiao Tong University; Renmin Hospital of Wuhan University: Wuhan University Renmin Hospital; Renmin Hospital of Wuhan University: Wuhan University Renmin Hospital

**Keywords:** COVID-19, SARS-CoV-2, endothelium, renal pathology, indicator

## Abstract

**Background:**

We aimed to analyse clinical characteristics and find potential factors predicting poor prognosis in patients with coronavirus disease 2019 (COVID-19).

**Methods:**

We analyzed the demographic and clinical data of COVID-19 patients and detected SARS-CoV-2 RNA in urine sediments collected from 53 COVID-19 patients enrolled in Renmin Hospital of Wuhan University from January 31, 2020 to February 18, 2020 with qRT-PCR analysis, and then classified those patients based on clinical conditions (severe or non-severe syndrome) and urinary SARS-CoV-2 RNA (U_RNA_^−^ or U_RNA_^+^).

**Results:**

We found that COVID-19 patients with severe syndrome (severe patients) showed significantly higher positive rate (11 of 23, 47.8%) of urinary SARS-CoV-2 RNA than non-severe patients (4 of 30, 13.3%, p = 0.006). U_RNA_^+^ patients or severe U_RNA_^+^ subgroup exhibited higher prevalence of inflammation and immune discord, cardiovascular diseases, liver damage and renal disfunction, and higher risk of death than U_RNA_^−^ patients. To understand the potential mechanisms underlying the viral urine shedding, we performed renal histopathological analysis on postmortems of patients with COVID-19 and found that severe renal vascular endothelium lesion characterized by increase of the expression of thrombomodulin and von Willebrand factor, markers to assess the endothelium dysfunction. We proposed a theoretical and mathematic model to depict the potential factors determining the urine shedding of SARS-CoV-2.

**Conclusions:**

This study indicated that urinary SARS-CoV-2 RNA detected in urine specimens can be used to predict the progression and prognosis of COVID-19 severity.

## Background

COVID-19 is a highly contagious disease caused by a newly emerging coronavirus called severe acute respiratory syndrome coronavirus 2 (SARS-CoV-2), which belongs to the β-coronavirus cluster that comprises 7 members, including SARS and Middle East Respiratory Syndrome (MERS) [1]. COVID-19 affects different people in different ways. SARS-CoV-2 infected patients have a wide range of symptoms reported – from mild symptoms to severe illness. In general, COVID-19 patients have usually demonstrated respiratory system symptoms, abnormal radiological images of chest computed tomography (CT) scan, and hematological changes [2]. Those patients under severe conditions might also be accompanied with multiple organ damages, such as on kidney, heart, digestive tract, blood and nervous system [3].

The main methods used for screening and diagnosis of SARS-CoV-2 infection include the detection of SARS-CoV-2 nucleic acid and SARS-CoV-2-specific antibody. The test for SARS-CoV-2 nucleic acid in nasopharyngeal swab specimens with reverse transcription-polymerase chain reaction (RT-PCR) was almost the only pathogen detection method used at the early stage of COVID-19 outbreak in Wuhan, China, and later this test was applied to other body fluid samples, such as anal swab, stool, blood and urine, to avoid false negative results. It has been demonstrated that the positive rates of SARS-CoV-2 nucleic acid in different body fluids are variable, indicating a distinct pattern of persistence and clearance of viral RNA in body fluids in COVID-19 patients [4-6]. The positive rates detected in extra-pulmonary specimens are usually lower than those detected in nasopharyngeal swabs, which may have special significance in evaluation of disease condition and determining the virus shedding routes [4, 5]. Similar to previous reports [4-13], we also found that urinary SARS-CoV-2 RNA could be detected in COVID-19 patients, indicating that under certain specific conditions SARS-CoV-2 might be infiltrated from blood stream to kidney parenchyma, and eventually resulted in renal injury and urinary shedding of viruses. Given the clinical features of relatively low rate of severe illness among COVID-19 patients and low prevalence of kidney involvement [3, 14-16], coexisting with a low urinary virus RNA positive rate in COVID-19 patients, we hypothesize that the detection of urinary SARS-CoV-2 nucleic acid, which may resulted in renal and cardiovascular endothelial destruction to facilitate the virus access to the kidney parenchyma, with improved method may be used as a specific biomarker to indicate the severity of COVID-19.

## Methods

### Study design and patients

From January 31, 2020 to February 18, 2020, a total of 53 patients who were diagnosed with COVID-19 at Renmin Hospital of Wuhan University were tested for SARS-CoV-2 nucleic acid in urine samples with quantitative reverse transcription-polymerase chain reaction (qRT-PCR) analysis. To reduce false negative results, we collected the urine sediment samples from those patients at the admission day case by case. Based on the results of urine SARS-CoV-2 nucleic acid testing, we divided those patients into two groups, including the urinary SARS-CoV-2 negative group (U_RNA_^−^, 38 cases) and positive group (U_RNA_^+^, 15 cases). We then conducted a retrospective study on those patients’ clinical characteristics, basic diseases and laboratory tests (Supplemental Fig. 1). The diagnosis of COVID-19 pneumonia was conducted by following the New Coronavirus Pneumonia Prevention and Control Guidance (5^th^ edition) published by the National Health Commission of China [17]. Our study was approved by the ethics committee of Renmin Hospital of Wuhan University (wdry2020-k064), the Ethics Commission of General Hospital of Central Theatre Command ([2020]017-1), and the Ethics Commission of Jinyintan Hospital (KY-2020-15.01). Written informed consent was waived by the Ethics Commission of the participated hospitals for emerging infectious diseases.

### Data collection

The data of epidemiological characteristics, clinical manifestation, radiology examination and laboratory examination were collected from the electronic medical records, and the laboratory examination included arterial blood gas test, myocardial infarction, heart failure, liver and kidney function, electrolytes, whole blood cell count, blood biochemistry, coagulation test, immune function and C-reactive protein. The illness conditions were assessed and defined as severe and non-severe type depending on the existence of respiratory dysfunction, in that the severe type was defined as that the oxygen saturation is less than 93% under resting status, or the arterial oxygen pressure (PaO_2_)/fraction of inspired oxygen (FiO_2_) ratio is less than 300 mmHg. We identified 30 cases as non-severe patients and 23 as severe patients (Supplemental Fig. 1). All data were reviewed by a team of trained physicians.

### Virological analysis

The SARS-CoV-2 virus in urine from the 53 COVID-19 patients was detected with quantitative RT-PCR analysis as previously described [18]. In brief, the urine sediments from participants were collected for SARS-CoV-2 test with the detection kit (Bioperfectus, Taizhou, China). The ORF1ab gene (nCovORF1ab) and the N gene (nCoV-NP) were used for qRT-PCR analysis according to the manufacturer’s instructions. Reaction mixture were prepared and qRT-PCR assay was then performed under the following conditions: incubation at 50 °C for 15 minutes and 95 °C for 5 minutes, 40 cycles of denaturation at 94°C for 15 seconds, and extending and collecting fluorescence signal at 55 °C for 45 seconds.

### Tissue sampling and processing

Kidney samples were obtained from autopsies of 4 severe COVID-19 patients with multi-organ failure, including acute kidney injury (AKI) that was examined and confirmed in a designated pathology laboratory.

Immunohistochemical staining was performed on kidney specimens from autopsy for thrombomodulin (TM), and von Willebrand factor (vWF) as previously described [19]. Briefly, the sections were incubated with primary anti-TM (Cat: 14318-1-AP, 1:100, rabbit IgG; Proteintech Group, USA), anti-vWF (Cat: 11778-1-AP, 1:100, rabbit IgG; Proteintech Group, USA), or rabbit-isotype antibody (control) (1:100; Dako) at 4°C overnight, followed by the incubation with the HRP-anti-Rabbit secondary antibodies for 1h at room temperature. Peroxidase activity was visualized with the DAB Elite kit (K3465, DAKO). Positive staining as brown coloration was viewed by a light microscope.

### Statistical Analysis

Continuous variables were expressed using the mean ± standard deviation (normal distribution) or medians and interquartile (IQR) values as appropriate (abnormal distribution). Categorical variables were shown as the percentages and counts. Two-independent group *t*-tests was used when the data were normally distributed, otherwise, wilcoxon rank-sum test was used. Chi-square tests and Fisher’s exact tests were applied to categorical variables as appropriate. The cumulative rate of in-hospital survival was investigated using the Kaplan-Meier method. All statistical analyses were performed using SPSS 22.0. *p* < 0.05 was considered as statistically significant.

## Results

### Characterization of patients with U_RNA_^+^ and U_RNA_^−^

A total of 53 hospitalized COVID-19 patients were enrolled in this study. The characteristics of those patients were detailed in Table 1. The median age of those patients was 52 years old (IQR, 42-66), and 58% of those patients were female. By testing SARS-CoV-2 nucleic acid in urine samples with qRT-PCR analysis, we found that 38 of those 53 patients were urinary SARS-CoV-2 negative (U_RNA_^−^) and 15 of them were urinary SARS-CoV-2 positive (U_RNA_^+^). The U_RNA_^+^ patients were older and more likely to experience chest tightness and shortness of breath than U_RNA_^−^ patients, but showed no significant differences in male/female distribution, fever, cough, sputum production, fatigue, radiological appearance, hypertension, diabetes, cardiovascular diseases, chronic renal disease (Table 1). In addition, U_RNA_^+^ patients suffered more severe respiratory distress with manifestations of lower arterial oxygen pressure (PaO_2_) and oxygen saturation (SaO_2_) than U_RNA_^−^ patients as examined with arterial blood gas analysis (Table 2). The leukopenia and lymphocytopenia were detected more frequently in routine blood test of U_RNA_^+^ patients than those in blood test of U_RNA_^−^ patients (Fig. 1a). Immune profile evaluation identified a more frequent increase of serum CRP and IgE in U_RNA_^+^ patients (Fig. 1b and c). In addition, we found that U_RNA_^+^ patients had higher prevalence of increased serum levels of ALT (Fig. 1d), higher percentage of decreased serum AST (Fig. 1e), higher case percentage of increased serum myoglobin, ultra-TnI (Fig. 1f and g), LDH (Fig. 1h), urea (Fig. 1i), and decreased eGFR (Fig. 1j) than U_RNA_^−^ patients. These data indicated that U_RNA_^+^ patients had more severe lesions on organs of liver, heart, and kidney. We further found that U_RNA_^+^ patients showed significantly lower levels of T cells and T helper (Th) cells (Fig. 2a and b) in peripheral blood mononuclear cells, and higher levels of serum CRP (Fig. 2c), ALT (Fig. 2d), but lower serum AST (Fig. 2e), and higher levels of DBIL (Fig. 2f), LDH (Fig. 2g), urea (Fig. 2h), and significantly lower eGFR (Fig. 2i) than U_RNA_^−^ patients. These results suggested that U_RNA_^+^ patients might develop severe clinical manifestations characterized by the increase of the levels of inflammatory markers and tissue injury-related enzymes.

### Clinical features and prognosis of severe patients with U_RNA_^+^ and U_RNA_^−^

Above results implied a correlation of the urinary SARS-CoV-2 RNA with COVID-19 disease severity and the underlying conditions in COVID-19 patients. We hypothesized that urinary SARS-CoV-2 RNA may serve as a biomarker for predicting the clinical outcomes of severe COVID-19 patients. In the total of 53 patients, we identified 30 non-severe patients and 23 severe patients (Supplemental Fig. 1) based on the oxygen saturation (less than 93% under resting status) and the arterial oxygen pressure (PaO_2_)/fraction of inspired oxygen (FiO_2_) ratio (less than 300 mmHg). Within the 23 severe patients, we found that 12 of those 23 server patients were urinary SARS-CoV-2 negative (SU_RNA_^−^) and 11 of them were U_RNA_^+^ (SU_RNA_^+^). The positive rate of urine SARS-CoV-2 RNA were significantly higher in sever patients (11/23 severe patients = 47.8%) than that in non-severe patients (4/30 non-severe patients = 13.3%) (Fig. 3a). This result suggested that urine shedding SARS-CoV-2 correlated with the severity of the disease. To support this notion, we found that SU_RNA_^+^ patients experienced more comorbidities, including higher prevalence of hypertension and cardiovascular diseases (Fig. 3b-d), and renal function impairment (Fig. 3e). SU_RNA_^+^ patients also showed significantly lower levers of eGFR (Fig. 3f) but higher levels of IgE and IgG (Fig. 3g and h) than SU_RNA_^−^ patients. In addition, the U_RNA_^+^ patients among 53 patients had a significantly higher risk of death than the U_RNA_^−^ patients (Fig. 4a). Furthermore, the severe patients with U_RNA_^+^ also demonstrated a higher risk of death than the severe patients with U_RNA_^−^, although the difference did not reach statistical significance between U_RNA_^−^ and U_RNA_^+^ groups of severe patients (Fig. 4b). These results suggested that U_RNA_^+^ patients exhibited significantly higher risk of cardiovascular abnormality, liver injury, renal disfunction and death, and urinary SARS-CoV-2 RNA might serve as a predictive biomarker for determining the prognosis and outcome of the disease.

### The expression of thrombomodulin (TM) and von Willebrand factor (vWF) was increased in renal tissues from died COVID-19 patients

Endothelial dysfunction is prevalent in chronical cardiovascular disease and infectious or inflammatory diseases (such as that in virus-infected patients) [20-26]. TM and vWF have been recognized as biomarkers to assess the endothelium dysfunction [20-24]. We found that the expression of TM and vWF in interstitial vessels, glomerular, and tubules were higher in kidneys from died COVID-19 patients compared to that in kidneys from renal carcinoma patients (Fig. 5), suggesting that renal vascular endothelial lesion might also associate with the severity of COVID-19 patients.

### Theoretical and mathematic modeling of urine shedding of SARS-CoV-2

SARS-CoV-2 mainly infects host through respiratory tract and spread to other organs or tissues through blood circulation. Damaged vascular endothelial, which either preexisted or incurred by virus infection, allows virus infiltration between blood stream and tissue mesenchyme by passing through endothelium. Virus-related renal insult and urine shedding are determined by multifactor, such as the virus load of inflow, the vascular endothelial integrity, and the intensity of anti-virus response. In this study, we adopt a model of renal Inflow-Infiltration-Injury-Into urine (“4I” model) to illustrate the course and outcome of SARS-CoV-2 infection in COVID-19 patient kidney (Fig. 6a). In this model, the kidney is simplified as an effector to response to the virus invasion (input) with the effects of renal damages and virus shedding (output). Output effects are determined by the input strength (amount of loading virus) and the intrinsic nature of the kidney, such as the integrity of vascular endothelium and local immunity (Fig. 6b). Although the compromised integrity of glomeruli and interstitial vascular vessels both allow the virus infiltration into kidney parenchyma, the infiltrating route through interstitial vessels is ignored, since the exuded virus is unlikely to infect tubular epithelial cells in which the ACE2 enriched in the brush border [27]. In that context, we propose a function equation and curve to solve the urinary excretion of viral nucleic acid, condition of vascular endothelial integrity and the circulating viral load in COVID-19 patients (Fig. 6c).

## Discussion

We conducted a comprehensive study on the clinical characteristics, basic diseases and laboratory tests in a cohort of 53 COVID-19 patients, where 30 patients had non-severe symptoms, and 23 patients suffered from severe symptoms. Our results suggested an intriguing association of the urinary SARS-CoV-2 RNA with the clinical manifestation of COVID-19, pointing to a high viral load, new or preexisting vascular endothelial damage in severe COVID-19 patients. This retrospective study indicated for the first time that the positive of SARS-CoV-2 RNA in urine specimens maybe used to predict the progression and prognosis of COVID-19.

SARS-CoV-2 infection and transmission in human has been successfully reproduced in ferret model, in which SARS-CoV-2 viruses can be detected in both kidney tissues and urine [28]. Viral infection and replication in kidney tissues indicate that the virus might be shed through urine. However, the positive rates of urine SARS-CoV-2 RNA vary from 0-7.5% (Table 3). The disparity in detection of the urinary SARS-CoV-2 RNA might be resulted in the false negative test of nucleic acid by qRT-PCR, which may be caused by inadequate sampling, low viral load, or other unknown factors [4, 5]. In the ferret model of SARS-CoV-2 infection, urinary SARS-CoV-2 RNA could be detected up to 8 days after the onset in the virus inoculated group in contrast to only 4 days in the directly contacted group [28], suggesting that the amount of viral loads and the stages of illness may have impact on the positive test rates. In addition, appropriate sampling seems to be essential for avoiding false negative results in COVID-19 patient samples. In our study, we optimized and analyzed the specimens of urine sediments, and found that 15 of those 53 patients were urinary SARS-CoV-2 positive (U_RNA_^+^) (Table 1). Our analysis showed a positive rate of 28.3% from 53 COVID-19 patients, which are higher than test results on routine urine sample testing (Table 3) [5, 7, 8, 10-13]. This result suggested that our optimized urine SARS-CoV-2 RNA test method could improve the positive rate, which may assist in predicting the disease outcome, especially in severe patients.

We also observed that U_RNA_^+^ patients or severe U_RNA_^+^ subgroup showed higher prevalence of inflammation and immune discord, cardiovascular diseases, liver damage and renal disfunction, and higher risk of death than U_RNA_^−^ patients. The reason for the observed higher prevalence of developing severe clinical manifestations and higher risk of death in U_RNA_^+^ patients might be caused by a high SARS-CoV-2 viral load, which was shown to to be highly associated with in-hospital mortality in COVID-19 patients [29]. The new or preexisting vascular endothelial damage in COVID-19 patients may also lead to the increase of inflammation and damage within infected tissue and the excretion of SARS-COV-2 into urine. Therefore, the evaluation of vascular endothelial damage may be an important prognostic tool to understand the outcomes of COVID-19 patients.

Studies on cellular mechanism have confirmed that SARS-CoV-2 shares the same membrane-bound angiotensinconverting enzyme 2 (ACE2) as SARS-CoV to gain access to its target cells [30-32]. In particular, kidneys show much more robust expression of ACE2 than respiratory organs, suggesting that kidney is a possible infecting target of SARS-CoV-2 [33]. The involvement of kidneys is usually evaluated in two aspects of renal functional impairment and renal insult. Acute renal injury (AKI) was rarely happened in COVID-19 patients with incidence of 3-9%, but herald very poor prognostic with very high mortality [3, 14]. Kidney insults manifestations of proteinuria (44%) and hematuria (26.7%), were commonly seen in COVID-19 infection [3, 14, 16]. Until recently, the renal involvement in COVID-19 patients remains a matter of widely concern and debate as one study suggested that the renal insults were uncommon in COVID-19 patients [9]. To resolve the difference and controversy, two major issues need to be addressed. The first one is whether SARS-CoV-2 is able to infect the target cells through blood flow to kidney mesenchyme, and the second one is whether the infection can cause detrimental effects to the kidney tissue. Previous study has showed that spherical virus-like particles characteristic of coronavirus was found in the renal tubular epithelium and podocytes [34]. Considering that the infection of SARS-CoV-2 will trigger severe inflammation, we further explored whether SARS-CoV-2 infection induced renal endothelium dysfunction. We found that the expression of TM and vWF, biomarkers of endothelium dysfunction, were increased in renal tissues from died COVID-19 patients (Fig. 5). This result supported that SARS-CoV-2 can cause detrimental effects on kidney tissues. The factors related to the severity of renal dysfunction in COVID-19 patients are not yet fully understood. It could potentially be caused by the amount of infiltrated virus in kidneys, which may be largely dependent on the inflow virus and the nature of the endothelium.

Our findings support a significant role of vascular endothelial lesion in COVID-19 patients in the development of renal damage and urine shedding. To further illustrate the course and outcome of SARS-CoV-2 infection in COVID-19 patient kidneys, we raised a model of renal Inflow-Infiltration-Injury-Into urine (“4I” model) (Fig. 6a). This model may help us to understand the role and function of kidneys in SARS-CoV-2 infection. Kidneys are simplified as an effector to response to the virus invasion (input) with the effects of renal damages and virus shedding (output). SARS-CoV-2-related renal insult and urine shedding may determine by multifactor, including the virus load of inflow, the vascular endothelial integrity, as well as the intensity of anti-virus response. Our analysis on the clinical features of the cluster showed that the positive test rate of urine SARS-CoV-2 RNA is remarkably higher in the severe patients than in non-severe patients, and renal vascular endothelial lesion associate with severity of COVID-19 patients. Based on these finding, we propose a function equation and curve to solve the urinary excretion of viral nucleic acid, condition of vascular endothelial integrity and the circulating viral load (Fig. 6c). The function equation and curve may provide another strategy to calculate and predict the progression and prognosis of the disease.

However, due to the limited sample size, our model might not be powered sufficiently to reflect the overall complexity of the general population. Therefore, large-scale prospective cohort studies are required in ethnically and geographically diverse cohorts to better understand the association and importance of U_RNA_^+^ in the disease progression of COVID-19. In addition, due to the lacking of clinical data of patients after discharge, we could not assess the association of urinary SARS-CoV-2 RNA with disease recovery. The precise relationship between urinary SARS-CoV-2 RNA and endothelial dysfunction and multiple organ disfunction in these patients require further investigation. Furthermore, our hypothesis about function equation and curve to solve the urinary excretion of viral nucleic acid, condition of vascular endothelial integrity and the circulating viral load should be tested in future studies.

## Conclusion

We optimized the method of urine SARS-CoV-2 RNA detection and significantly increased the positive detection rates. We analyzed the clinical characteristics of patients with urinary nucleic acid positive SARS-CoV-2 RNA, and revealed a potential association of vascular endothelial damage with virus urine shedding. Furthermore, we established a model to analyze the relationship between virus urine excretion and the underlying disease condition. This study suggests that the detection of SARS-CoV-2 RNA in urine sediments can provide a robust biomarker for evaluation and prognosis for patients with COVID-19.

## Supplementary Material

Supplement

## Figures and Tables

**Table 1.** Demographic and Clinical characteristics of 53 enrolled patients.

	All patients (n=53)	Urinary SARS-CoV- 2		P value	Illness severity				P value
		U_RNA_^−^ (n=38, 71.7%)	U_RNA_^+^ (n=15, 28.3%)	U_RNA_^−^ *vs* U_RNA_^+^	Non-severe		Severe		S U_RNA_^+^ *vs* S U_RNA_^−^
					U_RNA_^−^ (n=26, 86.7%)	U_RNA_^+^ (n=4, 13.3%)	U_RNA_^−^ (n=12, 52.2%)	U_RNA_^+^ (n=11, 47.8%)	
Demographic characteristic (No., %)									
Age, years	52 (42.0-66.0)	51.0 (38.3-59.8)	61.0 (48.5-72.0)	0.016	50.5 (37.3-56.8)	50.0 (48.5-60.8)	55.0 (51.0-63.5)	66.0 (50.0-72.0)	0.126
Female	31/53 (58%)	22/38 (58%)	9/15 (60%)	0.889	17/26 (65%)	3/4 (75%)	5/12 (42%)	6/11 (55%)	0.684
Respiratory symptoms (No., %)									
Fever	41/53 (77.4%)	31/38 (73.7%)	13/15 (86.7%)	0.969	16/26 (61.5%)	4/4 (100.0%)	12/12 (100.0%)	9/11 (81.8%)	0.217
Cough	38/53 (71.7%)	31/38 (73.7%)	10/15 (66.7%)	0.421	19/26 (73.1%)	3/4 (75.0%)	9/12 (75.0%)	7/11 (63.6%)	0.667
Sputum production	13/53 (24.5%)	9/38 (23.7%)	6/15 (40.0%)	0.396	5/26 (19.2%)	3/4 (75.0%)	2/12 (16.7%)	3/11 (27.3%)	0.640
Fatigue	18/53 (34.0%)	11/38 (29.0%)	7/15 (46.7%)	0.220	9/26 (34.6%)	3/4 (75.0%)	2/12 (16.7%)	4/11 (36.4%)	0.371
Chest tightness	14/53 (26.4%)	6/38 (15.8%)	9/15 (60.0%)	0.001	3/26 (11.5%)	3/4 (75.0%)	2/12 (16.7%)	6/11 (54.6%)	0.089
Shortness of breath	19/53 (35.9%)	12/38 (31.6%)	10/15 (66.7%)	0.02	0/26 (0.0%)	0/4 (0.0%)	9/12 (75.0%)	10/11 (90.9%)	0.590
Radiological appearance (No., %)									
Unilateral pneumonia	2/53 (3.77%)	2/38 (5.3%)	0/15 (0.0%)	0.365	2/26 (7.7%)	0/4 (0.0%)	0/12 (0.0%)	0/11 (0.0%)	UTC
Bilateral pneumonia	35/53 (66.0%)	26/38 (68.4%)	9/15 (60.0%)	0.560	17/26 (62.4%)	1/4 (25.0%)	9/12 (75.0%)	8/11 (72.7%)	1.000
Multiple “ground-glass opacity” lesions	20/35 (37.7%)	17/38 (44.7%)	3/15 (20.0%)	0.094	14/26 (53.9%)	0/4 (0.0%)	3/12 (25.0%)	3/11 (27.3%)	1
Comorbidities (No., %)									
Hypertension	19/53 (35.9%)	11/38 (29.0%)	8/15 (53.3%)	0.095	9/26 (34.6%)	0/4 (0.0%)	2/12 (16.7%)	8/11 (72.7%)	0.012
Diabetes	7/53 (13.2%)	5/38 (13.2%)	2/15 (13.3%)	0.986	3/26 (11.5%)	0/4 (0.0%)	2/12 (16.7%)	2/11 (18.2%)	1.000
Cardiovascular diseases	6/53 (11.3%)	2/38 (5.3%)	4/15 (26.7%)	0.083	2/26 (7.7%)	0/4 (0.0%)	0/12 (0.0%)	4/11 (36.4%)	0.037
Chronic renal disease	2/53 (3.8%)	2/38 (5.3%)	0/15 (0.0%)	0.365	2/26 (7.7%)	0/4 (0.0%)	0/12 (0.0%)	0/11 (0.0%)	UTC
In-hospital death (No., %)	6/53 (11.3%)	2/38 (5.3%)	4/15 (26.7%)	0.083	0/26 (0.0%)	0/4 (0.0%)	2/12 (16.7%)	4/11 (36.4%)	0.371

Data were showed as medians (interquartile ranges, IQR) and numbers/total (%); UTC, unable to calculate; U_RNA_^−^, negative urinary SARS-CoV-2; U_RNA_^+^, positive urinary SARS-CoV-2; S, Severe. P values presented the comparison between Negative cases and positive cases. *p* < 0.05 was considered statistically significant.

**Table 2.** Arterial blood gas analysis of 53 enrolled patients.

	All patients (n=53)	Urinary SARS-CoV-2		P value	Illness severity				P value
		U_RNA_^−^ (n=38)	U_RNA_^+^ (n=15)	U_RNA_^−^ *vs* U_RNA_^+^	Non-severe		Severe		S U_RNA_^+^ *vs* S U_RNA_^−^
					U_RNA_^−^ (n=26)	U_RNA_^+^ (n=4)	U_RNA_^−^ (n=12)	U_RNA_^+^ (n=11)	
Arterial blood gas (No., %)									
PO_2_ (<100)	18/53 (34.0%)	8/38 (21.1%)	10/15 (66.7%)	0.002	0/26 (0.0%)	0/4 (0.0%)	8/12 (66.7%)	10/11 (90.9%)	0.317
PO_2_ (<80)	13/53 (24.5%)	6/38 (15.8%)	7/15 (46.7%)	0.046	0/26 (0.0%)	0/4 (0.0%)	6/12 (50.0%)	7/11 (63.6)	0.680
PCO_2_ (>46)	9/53 (17.0%)	5/38 (13.2%)	4/15 (26.7%)	0.439	0/26 (0.0%)	0/4 (0.0%)	5/12 (41.7%)	4/11 (36.4%)	1.000
SaO_2_ (≤93)	23/53 (43.4%)	12/38 (31.6%)	11/15 (73.3%)	0.006	0/26 (0.0%)	0/4 (0.0%)	12/12 (100.0%)	11/11 (100.0%)	UTC

Data are showed as numbers/total (%), P values present the comparison between Negative cases and positive cases. The normal ranges of PO_2_, PCO_2_ and SaO_2_ are 80-100 mmHg, 35-45 mmHg and 95 %, respectively. UTC, unable to calculate; U_RNA_^−^, negative urinary SARS-CoV-2; U_RNA_^+^, positive urinary SARS-CoV-2; S, Severe.

**Table 3.** Comparison of urinary SARS-CoV-2 nucleic acid detection in literature reports

Authors	Sampling method	PCR	Positive rate	Target gene	Detection kit	Participants condition	Refs
Huiming Wang, et al	Urine sediments	RT-PCR	28.3%	NP and ORF1ab	Zhongzhi, Wuhan	30 non-severe 23 severe	This article
Luwen Wang, et al	Urine sediments	RT-PCR	7.5%	NP and ORF1ab	Zhongzhi, Wuhan	48 non-CKD, 5 CKD	[9]
Chaolin Huang, et al	Urine	RT-PCR	11%	NP and ORF1ab	ND	9 moderates	[2]
Hongzhou Lu, et al	Urine	RT-PCR	6.9%	NP and ORF1ab	Master Biotechnology, China	Recovered	[5]
Zhenglin Yang, et al	Urine	RT-PCR	0%	NP and ORF1ab	GeneoDx (GZ-TRM2, China), Maccura (Sichuan, China) and Liferiver (W-RR-0479-02, China)	5 Uncomplicated, 14 complicated	[7]
Barnaby Edward Young, et al	Urine	RT-PCR	0%	N, S, and ORF1ab	EZ1 virus mini kit v2.0Qiagen	6 mild, 4 severe	[8]
Roman Wölfel, et al	Urine	RT-PCR	0%	E- and RdRp	Tib-Molbiol,Berlin, Germany	mild	[10]
Yifei Chen, et al	Urine	RT-PCR	0%	NP and ORF1ab	ND	ND	[11]
Chin Ion Lei, et al	Urine	qRT-PCR	0%	NP and ORF1ab	BioGerm, China	2 mild, 4 moderates	[12]
Fujie Zhang, et al	Urine	RT-PCR and ddPCR	0%	NP and ORF1ab	Shanghai BioGerm Medical Technology Co. LTD, China (RT-PCR) TargetingOne, Beijing, China (ddPCR)	ND	[13]

ND: not determined

## Abbreviations

PaO_2_: arterial oxygen pressure
FiO_2_: fraction of inspired oxygen
TM: thrombomodulin
vWF: von Willebrand factor
SaO_2_: oxygen saturation
ALT: Alanine Aminotransferase
AST: Aspartate Aminotransferase
LDH: Lactic Acid Dehydrogenase
Egfr: Estimated Glomerular Filtration Rate
ACE2: Angiotensin converting enzyme-2
