# Supplementary figures and images for: Urinary SARS-CoV-2 RNA is An Indicator For The Progression and Prognosis of COVID-19 Disease
Source: Res Sq. 2021 Feb 18:rs.3.rs-203728. Preprint. [Version 1] doi: 10.21203/rs.3.rs-203728/v1 (PMC7899468; doi:10.21203/rs.3.rs-203728/v1)

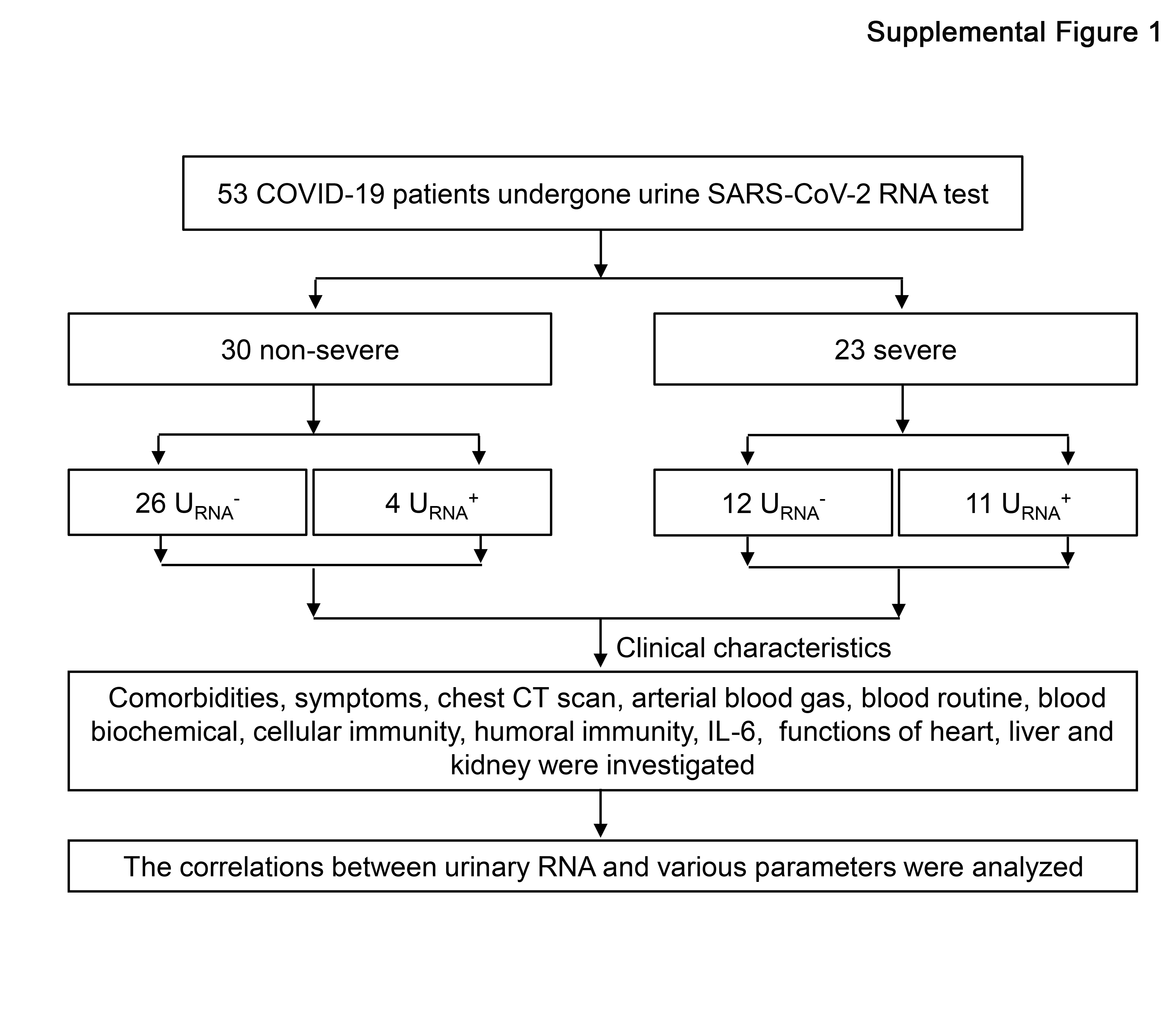

Supplement: Supplement [file 09a3471033053c3874e86fd9.tif]
